# The minimality of mean square error in chirp approximation using fractional fourier series and fractional fourier transform

**DOI:** 10.1038/s41598-022-23560-8

**Published:** 2022-11-10

**Authors:** Omar T. Bafakeeh, Muhammad Yasir, Ali Raza, Sami Ullah Khan, R. Naveen Kumar, M. Ijaz Khan, Deyab A. Almaleki, Nidhal Ben Khedher, Sayed M. Eldin, Ahmed M. Galal

**Affiliations:** 1 Department of Industrial Engineering, Jazan University, 82822 Jazan, Saudi Arabia; 2 Department of Mathematics, University of Engineering and Technology, Lahore, 54890 Pakistan; 3 Department of Mathematics, COMSATS University Islamabad, Sahiwal, 57000 Pakistan; 4 Department of Studies and Research in Mathematics, Davangere University, Davangere, Karnataka India; 5 Department of Mechanical Engineering, Lebanese American University, Beirut, Lebanon; 6 Umm Al-Qura University, P.O. Box 5555, 21955 Makkah, Saudi Arabia; 7 Department of Mechanical Engineering, College of Engineering, University of Hail, 81451 Hail, Saudi Arabia; 8 Laboratory of Thermal and Energy Systems Studies, National School of Engineering of Monastir, University of Monastir, 5000 Monastir, Tunisia; 9 Center of Research, Faculty of Engineering, Future University in Egypt New Cairo, New Cairo, 11835 Egypt; 10 Mechanical Engineering Department, College of Engineering, Prince Sattam Bin Abdulaziz University, Wadi Addawaser, 11991 Al-Kharj, Saudi Arabia; 11 Production Engineering and Mechanical Design Department, Faculty of Engineering, Mansoura University, Mansoura, P.O 35516 Egypt

**Keywords:** Engineering, Mathematics and computing

## Abstract

Chirps are familiar in nature, have a built-in resistance to noise and interference, and are connected to a wide range of highly oscillatory processes. Detecting chirp oscillating patterns by traditional Fourier series is challenging because the chirp frequencies constantly change over time. Estimating such types of functions considering the partial sums of a Fourier series in Fourier analysis does not permit an approximate solution, which entails more Fourier coefficients required for signal reconstruction. The standard Fourier series, therefore, has a poor convergence rate and is an inadequate approximation. In this study, we use a parameterized orthonormal basis with an adjustable parameter to match the oscillating behavior of the chirp to approximate linear chirps using the partial sums of a generalized Fourier series known as fractional Fourier series, which gives the best approximation with only a small number of fractional Fourier coefficients. We used the fractional Fourier transform to compute the fractional Fourier coefficients at sample points. Additionally, we discover that the fractional parameter has the best value at which fractional Fourier coefficients of zero degrees have the most considerable magnitude, leading to the rapid decline of fractional Fourier coefficients of high degrees. Furthermore, fractional Fourier series approximation with optimal fractional parameters provides the minimum mean square error over the fractional Fourier parameter domain.

## Introduction

Chirps often occur in nature because of the Doppler effect^1,2^. Chirps can be found^3–5^ both in nature, such as wave physics, mechanics, vibrations, biology, medicine^6^ Moreover, manufactured systems such as radar and sonar. The radar echo of a moving target with constant acceleration is a chirp function. In mathematics, chirps are often shown to exist in the forms of Weierstrass functions^7^ Riemann functions or Daubechies wavelets transforms of higher order.

A mathematical extension of the conventional Fourier series is the fractional Fourier series. Every subject where the traditional Fourier series is used has the potential to use it. The Fractional Fourier series is an elegant series with applications^8–10^ in engineering, science, and technology. Pei et al. ^10^ first introduced fractional Fourier series and used it in the expansion of linear chirps. Later, Barkat and Yingtuo^11^ presented modified fractional Fourier series that approximate generalized chirps with an arbitrary central frequency. Yu performed numerical simulations of Gaussian chirp signals^12^ using fractional Fourier series expansions. Coetmellec^13^ did fractional order Fourier analysis of chirped pulses and Brunel^14^ performed fractional order Fourier analysis of ultra-short pulse characterization and SPIDER interferograms. A fractional Fourier transform is a mathematical development of the conventional Fourier transform with a movable parameter, and it functions similarly to the conventional Fourier transform. In order to solve specific integro—differential equations and partial differential equations of quantum physics, Wiener developed the fractional Fourier transform^15^ in 1929. Bultheel^16^ constructed the mathematical theory of the fractional Fourier transformations by constructing its algebra and calculus. The theory of the fractional Fourier transform on the space $L_{2} \left( {\mathbb{R}} \right)$ was presented by McBride and Kerr^17^, and its implementations to partial differential equations were covered.

To sum up, the intention of this paper is twofold: First, to evaluate the fractional Fourier coefficients in closed form to provide the mathematical proofs of results related to chirp approximation, and second, to prove the minimality of mean square error in fractional Fourier series expansion of the linear chirps on fractional Fourier parameter domain. We outline this paper and provide a short overview of the main results. In "Preliminaries" section, we introduce chirps and exponential linear chirps. Then, we introduce fractional Fourier series and fractional Fourier transform for the space of square-integrable functions. In "Main results" section is the main section where we compute fractional Fourier coefficients by using the fractional Fourier transform method.

We also provide fresh findings about the fractional Fourier coefficients of linear chirps. We demonstrate that when the fractional Fourier parameter is fixed, the maximum value of the zero-degree fractional Fourier coefficient and the rapid decrease of the high-degree fractional Fourier coefficient are obtained. As a result, the approximation of the fractional Fourier series has a minimal mean square error. We summarize the findings and recommendations in "Conclusion" section.

## Preliminaries

A chirp function is a function that advances in time while sweeping all frequencies across a predetermined interval. A chirp can sweep frequencies in various ways (linear, quadratic, logarithmic, etc.). However, the most used chirps are linear.

### Definition

Chirps are functions defined by.

$$u = F\left( x \right)e^{{\iota {\Phi }\left( {\text{x}} \right)}}$$

where $F, {\Phi }$ are amplitude and phase functions, respectively. Such that $F\left( x \right), {\Phi }\left( x \right) \in {\mathbb{R}}$.

When compared to a smooth amplitude function, $F\left( x \right),$ with slow variations, the phase function ${\Phi }\left( x \right)$ is highly oscillatory, the variations of ${\Phi }\left( x \right)$ and $F\left( x \right)$ depend upon the following two conditions^18^ given below,

$$\left| {\frac{{\dot{F} \left( x \right)}}{{F\left( x \right) {\dot{\Phi }}\left( x \right){ }}}} \right| < < 1\;\;\;\;{\text{and}}\;\;\;\;\left| {\frac{{\ddot{\Phi } \left( x \right)}}{{\dot{\Phi }^{2} \left( x \right){ }}}} \right| < < 1,$$

where ${\dot{\Phi }}\left( {\text{x}} \right) \ne 0$. Both requirements given above are intended to define the concept of rapid oscillations within a slowly shifting envelope.

Since the human perception of sound intensity is depicted as a logarithmic function. By replacing the phase function $F\left( x \right)$ in the above equation with the function of the form

$$F\left( x \right) = e^{{ - \nu \pi x^{2} }} , \nu > 0$$

and amplitude function $\Phi \left( x \right)$ by a quadratic polynomial of the form

$$\Phi \left( x \right) = - \pi \lambda x^{2} , \lambda \in {\mathbb{R}}\backslash \left\{ 0 \right\}$$

the chirp becomes an exponential linear chirp, which is given below

$$u = e^{{ - \pi \nu x^{2} }} e^{{ - \iota \pi \lambda x^{2} }}$$

We consider the space $L_{2} \left[ { - \frac{T}{2},\frac{T}{2}} \right]$ of square-integrable complex-valued functions *u* that satisfy

$$\mathop \int \limits_{{ - \frac{T}{2}}}^{\frac{T}{2}} \left| u \right|^{2} dt < \infty$$

We establish the inner product between both the two complex-valued functions *u* and *v* in the range $L_{2} \left[ { - \frac{T}{2},\frac{T}{2}} \right]$ as follows

$$u,v_{{L_{2} }} = \mathop \int \limits_{{ - \frac{T}{2}}}^{\frac{T}{2}} u \overline{v}dx,$$

where $\overline{v}$ is the complex conjugate of *v*. Moreover, the norm *u* to be associated with is

$$u_{{L_{2} }} = \sqrt {\mathop \int \limits_{{ - \frac{T}{2}}}^{\frac{T}{2}} \left| u \right|^{2} } dx = \sqrt {u,u}$$

The fractional Fourier Transform of $u$ is defined as

$${\mathcal{F}}_{\theta } \left( j \right) = \mathop \int \limits_{ - \infty }^{\infty } j K_{\theta } \left( {x,j} \right)dx$$

where $\theta \in {\mathbb{R}}$ is the fractional Fourier order and $K_{\theta } \left( {x,j} \right)$ is the kernel of transformation, defined by

$$K_{\theta } \left( {x,j} \right) = \left\{ {\begin{array}{*{20}l} {\sqrt {\frac{1 - \iota \cot \theta }{{2\pi }}} e^{{\iota \left( {\frac{{x^{2} + u^{2} }}{2}} \right)\cot \theta - \iota j x\csc \theta }} } \hfill & {if\;\theta \ne n\pi } \hfill \\ {\delta \left( {x - j} \right)} \hfill & {\theta = 2n\pi } \hfill \\ {\delta \left( {x + j} \right)} \hfill & {\theta + \pi = 2n\pi } \hfill \\ \end{array} } \right.$$

where $\delta$ is the Dirac delta function.

The function *u* can be retrieved by applying inverse fractional Fourier transform defined as

$$u = \mathop \int \limits_{ - \infty }^{\infty } {\mathcal{F}}_{\theta } \left( j \right)K_{ - \theta } \left( {x,j} \right)dj.$$

If ${\varvec{\theta}} = 0$ the fractional Fourier transform of the function $u\left( x \right)$ is signal is itself if ${\varvec{\theta}} = \frac{{\varvec{\pi}}}{2}$ it becomes Fourier transform. The reconstruction of the chirp signal to minimize the error by finding the best fractional Fourier parameter is increasing. We introduce the fractional Fourier series for the space of $L_{2} \left[ { - \frac{T}{2},\frac{T}{2}} \right]$ The standard Fourier basis is replaced by the basis containing the chirp signal.

### Definition

Let the fractional Fourier basis for the function $u \in L_{2} \left[ { - \frac{T}{2},\frac{T}{2}} \right]$ is defined by.

$$\left\{ {\varphi_{n} ,_{\theta } \left( x \right):n \in {\mathbf{\mathbb{Z}}}} \right\},$$

where

$$\varphi_{n} ,_{\theta } \left( x \right) = \sqrt {\frac{\sin \theta + \iota \cos \theta }{T}} e^{{ - \iota \cot \theta \left[ {\frac{{x^{2} }}{2} + \frac{{1}}{2}\left( {n\frac{2\pi }{T}\sin \theta } \right)^{2} } \right] + \iota \frac{2\pi }{T}n x}}$$

on $\left[ { - \frac{T}{2},\frac{T}{2}} \right].$ Let $0 \le \theta \le \frac{\pi }{2}$ be the fractional Fourier domain. The fractional Fourier coefficients $\left\{ {C_{n,\theta } :n \in {\mathbf{\mathbb{Z}}}, 0 \le \theta \le \frac{\pi }{2}} \right\}$ of *u* computed by taking the inner product of chirp basis functions.

$\varphi_{n,\theta } \left( x \right)$ and chirp $u$ as

$$C_{n,\theta } = \mathop \int \limits_{{ - \frac{T}{2}}}^{\frac{T}{2}} u \overline{{\varphi_{n,\theta } \left( x \right)}} dx$$

The fundamental chirp function $\varphi_{n,\theta } \left( x \right)$ Fourier series is therefore described by

$$u = \mathop \sum \limits_{{n \in {\mathbf{\mathbb{Z}}}}} C_{n,\theta } \varphi_{n,\theta } \left( x \right).$$

The traditional Fourier series is a particular case of fractional Fourier series for $\theta = \frac{\pi }{2}$.

## Main results

The fractional Fourier coefficients are integrals with integrands as quadratic exponentials over a finite interval. Therefore, the fractional Fourier series on a finite interval is dealt with through numerical methods^10–13,19^. Fractional Fourier coefficients can be represented in the form of complex error functions. The error functions are special functions of mathematical physics that do not have any closed form. It is not straightforward to prove results mathematically. That is why the mathematical results on fractional Fourier series over a finite interval have been less available in the literature. We use the fractional Fourier transform method, which has introduced by S.C Pei^10^ to evaluate fractional Fourier coefficients.

### Lemma 1

Consider a linear chirp $u = e^{{ - \pi \nu x^{2} }} e^{{ - \iota \pi \lambda x^{2} }}$. Then the absolute value of fractional Fourier coefficients is.

$$\left| {C_{n,\theta } } \right| = \frac{\pi }{T}\frac{{e^{{\frac{{ - 2\pi^{3} n^{2} \nu }}{{T^{2} \left( {\pi^{2} \nu^{2} + \frac{{1}}{4}\left( {2\pi \lambda - \cot \theta } \right)^{2} } \right)}}}} }}{{\sqrt {\pi^{2} \nu^{2} + \frac{{1}}{4}\left( {2\pi \lambda - \cot \theta } \right)^{2} } }}$$

### Proof

The fractional Fourier coefficients for the chirp $u(x)$ are derived from the values obtained of the fractional Fourier series^10^.

$$C_{n.\theta } = \sqrt {\frac{2\pi \sin \theta }{T}} {\mathcal{F}}_{\theta } \left( {n\frac{2\pi }{T}\sin \theta } \right)$$

Using the definition of fractional Fourier transform, we have

$$C_{n,\theta } = \sqrt {\frac{2\pi \sin \theta }{T}} \mathop \int \limits_{ - \infty }^{\infty } e^{{ - \pi \nu x^{2} }} e^{{ - \iota \pi \lambda x^{2} }} K_{\theta } \left( {x,\frac{2n\pi }{T}\sin \theta } \right)dx$$

Replacing $u\left(x\right)$ and ${K}_{\theta }\left(x,\frac{2n\pi }{T}\mathrm{sin}\theta \right),$ we write ${C}_{n,\theta }$ as

3.1 $$C_{n,\theta } = \sqrt {\frac{2\pi \sin \theta }{T}} \mathop \int \limits_{ - \infty }^{\infty } e^{{ - \pi \nu x^{2} }} e^{{ - \iota \pi \lambda x^{2} }} \sqrt {\frac{1 - \iota \cot \theta }{{2\pi }}} e^{{ - \iota \cot \theta \left[ {\frac{{x^{2} }}{2} + \frac{{1}}{2}\left( {n\frac{2\pi }{T}\sin \theta } \right)^{2} } \right] + \iota \frac{2\pi }{T}n x}} dx$$

Simplification and collecting like terms, we write (3.1)

3.2 $$C_{n,\theta } = \sqrt {\frac{\sin \theta - \iota \cos \theta }{T}} e^{{\iota \left( {\frac{{n^{2} \sin \theta }}{{T^{2} }}} \right)}} \mathop \int \limits_{ - \infty }^{\infty } e^{{\left( { - \pi \nu - \iota \pi \lambda + \frac{\iota }{2}\cot \theta } \right)x^{2} - \iota n\frac{2\pi }{T}x}} dx$$

For sake of simplicity, we take

$$B = \sqrt {\frac{\sin \theta - \iota \cos \theta }{T}} e^{{\iota \left( {\frac{{n^{2} \sin \theta }}{{T^{2} }}} \right)}} , r = \pi \nu , s = \pi \lambda - \frac{{1}}{2}\cot \theta , and t = n\frac{2\pi }{T}.$$

Then (3.2) can be written in the simple form

3.3 $$C_{n,\theta } = B\mathop \int \limits_{ - \infty }^{\infty } e^{{ - \left( {r + \iota s} \right)x^{2} - \iota tx}} dx$$

Applying completing square method to the exponent of the integrand in (3.3)

3.4 $$C_{n,\theta } = Be^{{\frac{{ - t^{2} }}{{4\left( {r + \iota s} \right)}}}} \mathop \int \limits_{ - \infty }^{\infty } e^{{ - \left( {x\sqrt {r + \iota s} + \frac{\iota t}{{2\sqrt {r + \iota s} }}} \right)^{2} }} dx$$

We apply the substitution method to compute the integral. Let

$$\eta = x\sqrt {r + \iota s} + \frac{\iota t}{{2\sqrt {r + \iota s} }}. Then dx = \frac{d\eta }{{\sqrt {r + \iota s} }}$$

Now integral (3.4) becomes,

$$C_{n,\theta } = B\frac{{e^{{\frac{{ - t^{2} }}{{4\left( {r + \iota s} \right)}}}} }}{{\sqrt {r + \iota s} }} \mathop \int \limits_{ - \infty }^{\infty } e^{{ - \eta^{2} }} d\eta = Be^{{\frac{{ - t^{2} }}{{4\left( {r + \iota s} \right)}}}} \sqrt {\frac{\pi }{r + \iota s}.}$$

Since $C_{n, \theta }$ is a complex-valued, we use the property of complex-valued functions to write

3.5 $$\left| {C_{n,\theta } } \right|^{2} = \frac{{\left| B \right|^{2} \pi }}{{\sqrt {r^{2} + s^{2} } }}e^{{\frac{{ - rt^{2} }}{{2\left( {r^{2} + s^{2} } \right)}}}} ,$$

Since

$$\left| B \right|^{2} = \sqrt {\frac{{\sin^{2} \theta + \cos^{2} \theta }}{{T^{2} }}} = \frac{{1}}{T} and r^{2} + s^{2} = \pi^{2} \nu^{2} + \frac{{1}}{4}\left( {2\pi \lambda - \cot \theta } \right)^{2} .$$

Substituting $r, s, t and B$ in **(3.5)**, we have

3.6 $$\left| {C_{n,\theta } } \right|^{2} = \frac{\pi }{T}\frac{{e^{{\frac{{ - 2\pi^{3} n^{2} \nu }}{{T^{2} \left( {\pi^{2} \nu^{2} + \frac{{1}}{4}\left( {2\pi \lambda - \cot \theta } \right)^{2} } \right)}}}} }}{{\sqrt {\pi^{2} \nu^{2} + \frac{{1}}{4}\left( {2\pi \lambda - \cot \theta } \right)^{2} } }}$$

**Figure 1:**
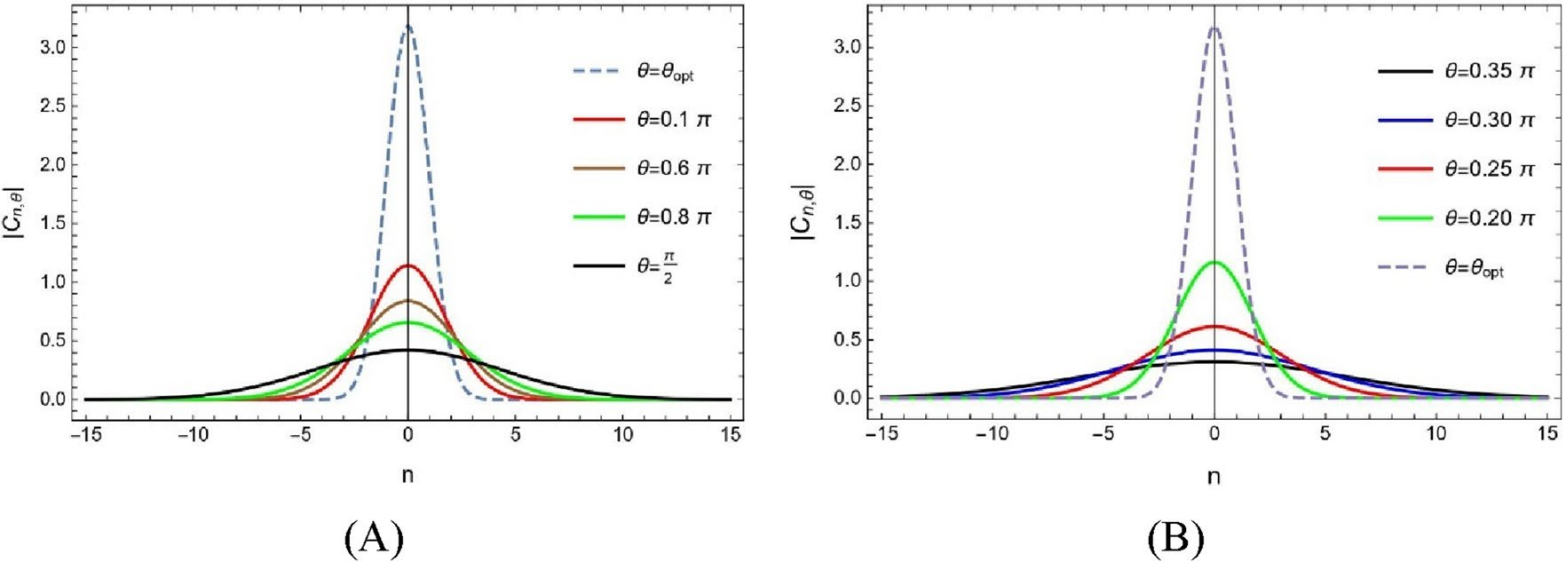
For the chirp $$u = e^{{ - \nu \pi x^{2} }} e^{{ - \iota \pi \lambda x^{2} }} T$$ he graphs of the $$\left| {C_{n,\theta } } \right|$$ using $$\lambda = 0.75$$ over $$- 15 \le n \le 15$$ (**A**) over the domain $$0 \le \theta \le \theta_{opt} \approx 0.209$$ (**B**) For $$0.209 \approx \theta_{opt} \le \theta \le \frac{\pi }{2}$$$$.$$

We use an optional fractional Fourier parameter $\theta_{opt}$ given by

$$\theta_{opt} = \tan^{ - 1} \left( {\frac{{1}}{2\pi \lambda }} \right)$$

in the $0 \le \theta \le \frac{\pi }{2}$ the domain of fractional Fourier parameters. Using the $\theta_{opt}$ parameter for the fractional Fourier transform. The fractional Fourier coefficient of zero degrees has an absolute value that is of the most significant magnitude. As a result, there is a rapid decline in the absolute amount of the fractional Fourier coefficients of great degree. Figure 1 depicts the behaviour of fractional Fourier coefficients $\left| {C_{n,\theta } } \right|$ for different fractional Fourier parameters.

**Figure 2:**
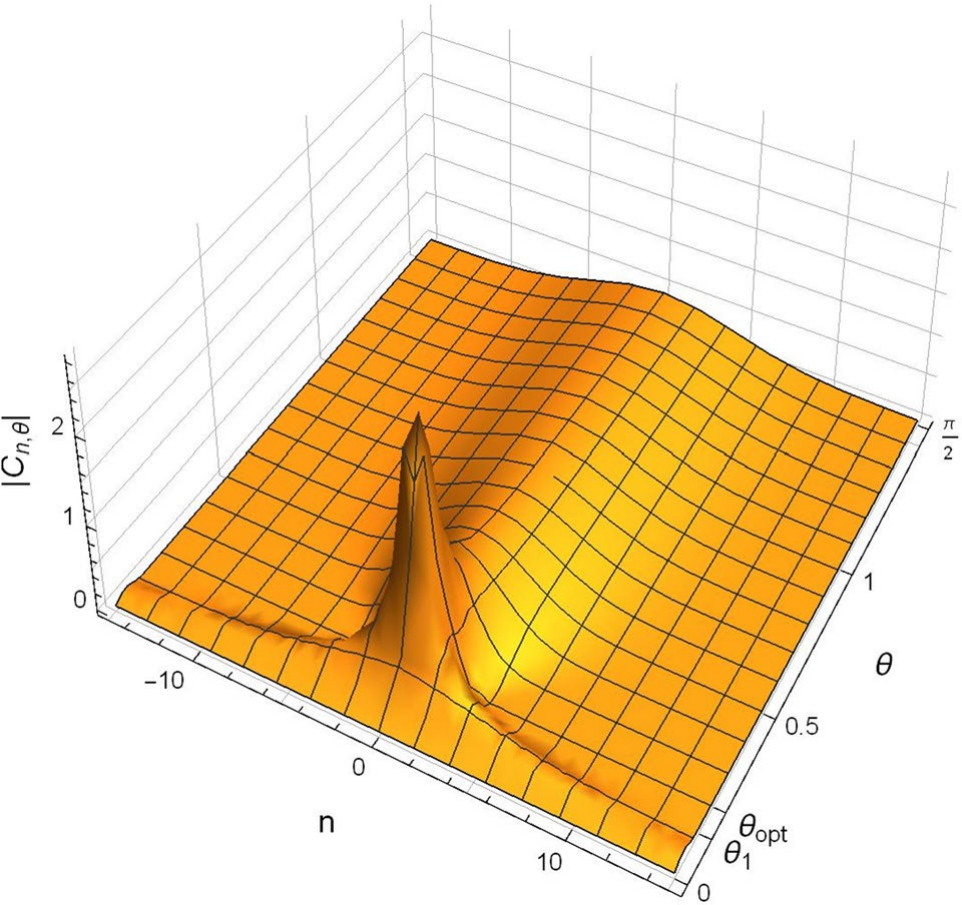
For the chirp $u = e^{{ - \nu \pi x^{2} }} e^{{ - \iota \pi \lambda x^{2} }} ,$ the three-dimensional plot of $\left| {C_{n,\theta } } \right|$, taking $\nu = 0.1\pi , \lambda = \frac{3}{4}$ over $- 15 \le n \le 15$.

The three-dimensional behavior of fractional Fourier coefficients $\left| {C_{n,\theta } } \right|$ fractional Fourier parameter domain $0 \le \theta \le \frac{\pi }{2}$ is shown in Fig. 2.

Theorem 1 establishes that the absolute value of fractional zero-degree Fourier coefficients has the most significant magnitude.

### Theorem 1

Consider a linear chirp $u = e^{{ - \nu \pi x^{2} }} e^{{ - \iota \pi \lambda x^{2} }} ,$ then zero-degree fractional Fourier coefficients have maximum value. Mathematically.

$$\arg \mathop {\max }\limits_{{n \in {\mathbb{\mathbf{Z}}}}} \left| {C_{n,\theta } } \right| = 0$$

### Proof

We prove the theorem for $n \in {\mathbb{R}}$. Since ${\mathbb{Z}} \subseteq {\mathbb{R}}$, the result holds for $n \in {\mathbb{Z}}$. Let.

$$U\left( n \right) = \left| {C_{n,\theta } } \right|^{2}$$.

Then by Lemma 1, we write

3.7 $$U\left( n \right) = \frac{\pi }{T}\frac{{e^{{\frac{{ - 2\pi^{3} n^{2} \nu }}{{T^{2} \left( {\pi^{2} \nu^{2} + \frac{{1}}{4}\left( {2\pi \lambda - \cot \theta } \right)^{2} } \right)}}}} }}{{\sqrt {\pi^{2} \nu^{2} + \frac{{1}}{4}\left( {2\pi \lambda - \cot \theta } \right)^{2} } }}$$

It is sufficient to prove.

$$\frac{dU}{{dn}} = 0$$ for $$n = 0$$.

$$\frac{dU}{{dn}} < 0$$ for $$n > 0$$.

$$\frac{dU}{{dn}} > 0$$ for $$n < 0$$.

Differentiating $U\left( n \right)$ in (3.7) concerning “n”, we have

$$\frac{dU}{{dn}} = \frac{\pi }{T}\frac{{e^{{\frac{{ - 2\pi^{3} n^{2} \nu }}{{T^{2} \left( {\pi^{2} \nu^{2} + \frac{{1}}{4}\left( {2\pi \lambda - \cot \theta } \right)^{2} } \right)}}}} }}{{\sqrt {\pi^{2} \nu^{2} + \frac{{1}}{4}\left( {2\pi \lambda - \cot \theta } \right)^{2} } }}\left( {\frac{{ - 2\pi^{3} \nu }}{{T^{2} \left( {\pi^{2} \nu^{2} + \frac{{1}}{4}\left( {2\pi \lambda - \cot \theta } \right)^{2} } \right)}}} \right)\left( {2n} \right)$$

Now setting

$$\frac{dU}{{dn}} = 0$$

we write.

3.8 $$\frac{\pi }{T}\frac{{e^{{\frac{{ - 2\pi^{3} n^{2} \nu }}{{T^{2} \left( {\pi^{2} \nu^{2} + \frac{{1}}{4}\left( {2\pi \lambda - \cot \theta } \right)^{2} } \right)}}}} }}{{\sqrt {\pi^{2} \nu^{2} + \frac{{1}}{4}\left( {2\pi \lambda - \cot \theta } \right)^{2} } }}\left( {\frac{{ - 2\pi^{3} \nu }}{{\pi^{2} \nu^{2} + \frac{{1}}{4}\left( {2\pi \lambda - \cot \theta } \right)^{2} }}} \right)\left( {2n} \right) = 0$$

From **(3.8)**, we see that $n = 0$. Here we conclude that $\frac{dU}{{dn}} > 0$ for $n < 0$ and $\frac{dU}{{dn}} < 0$ for $n > 0$.

We have proved that the absolute value of the fractional Fourier coefficient of zero degrees has maximum magnitude. Now we prove in Theorem 2 to show that the maximum absolute value of the fractional Fourier coefficient occurs when $\theta = \theta_{opt}$.

### Theorem 2

Consider a linear chirp $u = e^{{ - \nu \pi x^{2} }} e^{{ - \iota \pi \lambda x^{2} }}$. The zero-degree fractional Fourier coefficient has a maximum value when $\theta = \theta_{opt}$.

. i.e.,

$$\mathop {{\text{argmax}}}\limits_{{0 < \theta \le \frac{\pi }{2}}} \left| {C_{0,\theta } } \right| = \theta_{opt}\; where \;\theta_{opt} = \tan^{ - 1} \left( {\frac{{1}}{2\pi \lambda }} \right).$$

### Proof

Let.

$$V\left( \theta \right) = \left| {C_{0,\theta } } \right|^{2}$$

From Lemma 1, taking $n = 0$ in (3.6), we have

3.9 $$V\left( \theta \right) = \frac{\pi }{T}\frac{1}{{\sqrt {\pi^{2} \nu^{2} + \frac{{1}}{4}\left( {2\pi \lambda - \cot \lambda } \right)^{2} } }}$$

It is sufficient to prove.

$$\frac{dV}{{dn}} = 0$$ for $$\theta = \theta_{opt}$$.

$$\frac{dV}{{dn}} < 0$$ for $$\theta > \theta_{opt}$$.

$$\frac{dV}{{dn}} > 0$$ for $$\theta < \theta_{opt}$$.

Differentiating $V\left( \theta \right)$ in (3.9) with respect to $\theta$,

3.10 $$\frac{dV}{{d\theta }} = \frac{\pi }{T} \left( { - \frac{{1}}{4}\frac{{\left( {2\pi \lambda - \cot \lambda } \right)\csc^{2} \theta }}{{\left( {\pi^{2} \nu^{2} + \frac{{1}}{4}\left( {2\pi \lambda - \cot \theta } \right)^{2} } \right)^{\frac{3}{2}} }}} \right)$$

Now setting

$$\frac{dV}{{d\theta }} = 0$$

We have

3.11 $$\frac{\pi }{T} \left( { - \frac{{1}}{4}\frac{{\left( {2\pi \lambda - \cot \theta } \right)\csc^{2} \theta }}{{\left( {\pi^{2} \nu^{2} + \frac{{1}}{4}\left( {2\pi \lambda - \cot \theta } \right)^{2} } \right)^{\frac{3}{2}} }}} \right) = 0$$

From (3.11), we see that

$$\left( {2\pi \lambda - \cot \theta } \right)\csc^{2} \theta .$$

Since $\csc^{2} \theta \ne 0$ on $0 \le \theta \le \frac{\pi }{2}$. Therefore

$$\left( {2\pi \lambda - \cot \theta } \right) = 0 {\varvec\,{ or }\,} \theta = \tan^{ - 1} \frac{{1}}{2\pi \lambda } = \theta_{opt} .$$

$$\frac{dV}{{d\theta }} < 0 {\varvec\,{when}\,} 2\pi \lambda - \cot \theta > 0 \user2\,{ or }\, \theta > \tan^{ - 1} \left( {\frac{{1}}{2\pi \lambda }} \right) = \theta_{opt}$$

Now from (3.10), we see that

$$\frac{dV}{{d\theta }} > 0 {\varvec\,{when}\,} 2\pi \lambda - \cot \theta < 0 \user2\,{or }\, \theta < \tan^{ - 1} \left( {\frac{{1}}{2\pi \lambda }} \right) = \theta_{opt}$$

The minimal mean square error is produced by the partial sums of fractional Fourier series with the best possible fractional Fourier parameters while approximating linear chirps. The following sentence describes the best approximation: Think about a linear chirp

$$u = e^{{ - \nu \pi x^{2} }} e^{{ - \iota \pi \lambda x^{2} }} ,$$

the complex exponentials $\varphi_{n,\theta }$ and the $L_{2}$ norm $\left\| { \cdot_{L2} } \right\|$. We take all basis $\left\{ {\varphi_{n,\theta } :n \in {\mathbb{Z}}} \right\}$ with $0 \le \theta \le \frac{\pi }{2}$ and from the corresponding partial sums $S_{N,\theta } ,$ where

$$S_{N,\theta } \left( x \right) = \mathop \sum \limits_{\left| n \right| \le N} C_{n,\theta } \left( x \right)\varphi_{n,\theta } \left( x \right)$$

**Figure 3:**
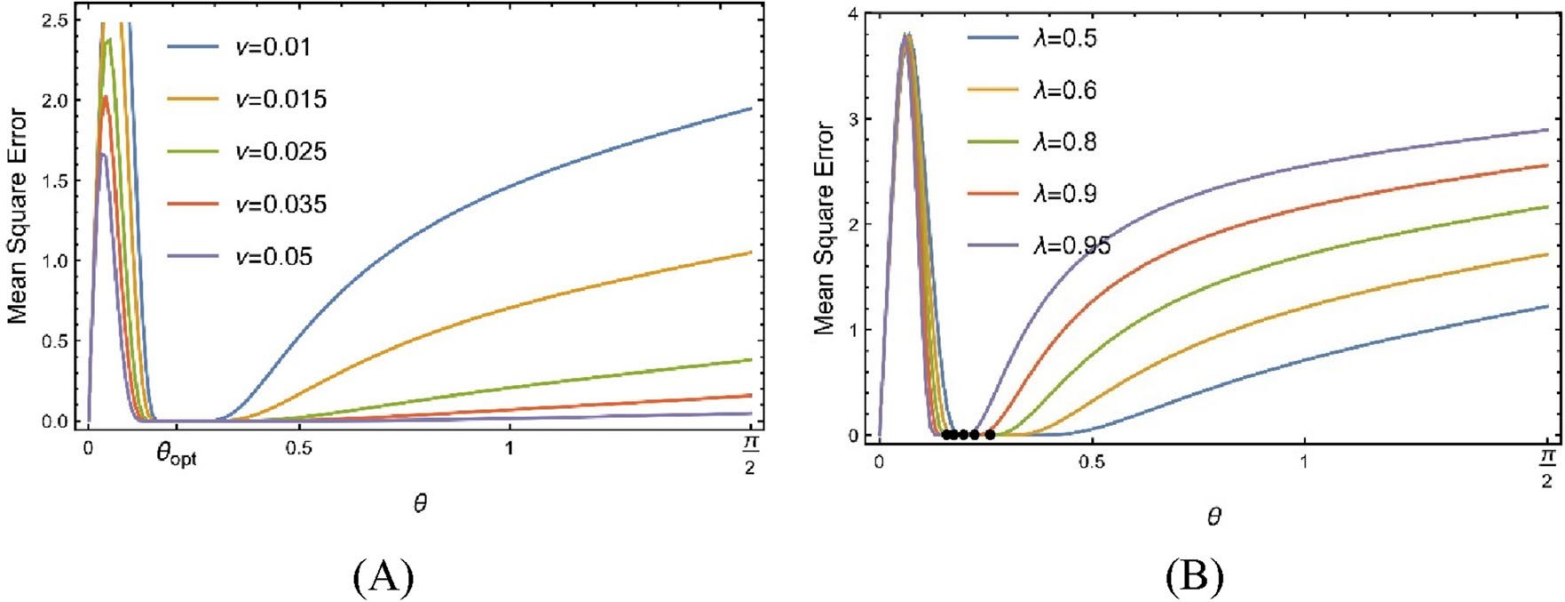
For the chirp $u = e^{{ - \nu \pi x^{2} }} e^{{ - \iota \pi \lambda x^{2} }} ,$ the graph of mean square error in the fractional Fourier series approximation of exponential chirps for $N = 3$ and (**A**) by taking $\lambda = 0.75$ for various values of $\nu$ (**B**) by taking $\nu = \frac{1}{200\pi }$ for various values of $\lambda$.

The partial sum $S_{N,\theta }$ is the best match to *u* on $\left[ { - \frac{T}{2},\frac{T}{2}} \right]$ for high N thanks to a parameter $\theta_{opt}$ in the fractional Fourier parameter domain $0 \le \theta \le \frac{\pi }{2}$. The fractional Fourier series estimate of chirps' mean square error is depicted in Fig. 3

We prove Theorem 3 to show that mean square error attains the minimum value at $\theta = \theta_{opt}$ when linear chirp is approximated by fractional Fourier series for sufficiently large values of $N$.

### Theorem 3

Consider a linear chirp $u = e^{{ - \nu \pi x^{2} }} e^{{ - \iota \pi \lambda x^{2} }}$. Then mean square error in fractional Fourier series approximation holds the following property.

$$\arg \min _{{0 \le \theta \le \frac{\pi }{2}}} \left\| {u - S_{{N,\theta }} } \right\|_{{L_{2} }} = \theta_{{opt}} ~~where~~~\theta_{{opt}} = \tan ^{{ - 1}} \left( {\frac{{1}}{{2\pi \lambda }}} \right).$$

### Proof

By using Parseval's identity, we write.

$$u - S_{{N,\theta L_{2} }} = \mathop \sum \limits_{{\left| {n \ge N + 1} \right|}} \left| {C_{n,\theta } } \right|^{2}$$

Let

$$Y\left( \theta \right) = \left| {C_{n,\theta } } \right|^{2}$$

From Lemma 1, using value of $\left| {C_{n,\theta } } \right|^{2}$, we express $Y\left( \theta \right)$ in the following form

3.12 $$Y\left( \theta \right) = \frac{\pi }{T}\frac{{e^{{\frac{{ - 2\pi^{3} n^{2} \nu }}{{T^{2} \left( {\pi^{2} \nu^{2} + \frac{{1}}{4}\left( {2\pi \lambda - \cot \theta } \right)^{2} } \right)}}}} }}{{\sqrt {\pi^{2} \nu^{2} + \frac{{1}}{4}\left( {2\pi \lambda - \cot \theta } \right)^{2} } }}$$

It is sufficient to prove.

$$\frac{dY}{{d\theta }} = 0$$ for $$\theta = \theta_{opt}$$.

$$\frac{dY}{{d\theta }} > 0$$ for $$\theta > \theta_{opt}$$.

$$\frac{dY}{{d\theta }} < 0$$ for $$\theta < \theta_{opt}$$.

Differentiating $Y\left( \theta \right)$ in (3.12) concerning $\theta$, we get

$$\frac{dY}{{d\theta }} = \frac{\pi }{T} \frac{{e^{{\frac{{ - 2\pi^{3} n^{2} \nu }}{{T^{2} \left( {\pi^{2} \nu^{2} + \frac{{1}}{4}\left( {2\pi \lambda - \cot \theta } \right)^{2} } \right)}}}} \left( {2\pi \lambda - \cot \theta } \right)\csc^{2} \theta }}{{\left( {\pi^{2} \nu^{2} + \frac{{1}}{4}\left( {2\pi \lambda - \cot \theta } \right)^{2} } \right)^{\frac{3}{2}} }}\left[ { - \frac{{1}}{4} + \frac{{\pi^{3} n^{2} \nu }}{{T^{2} \left( {\pi^{2} \nu^{2} + \frac{{1}}{4}\left( {2\pi \lambda - \cot \theta } \right)^{2} } \right)}}} \right]$$

After simplification of like terms, we have

$$\frac{dY}{{d\theta }} = \frac{\pi }{{4T^{3} }} \frac{{e^{{\frac{{ - 2\pi^{3} n^{2} \nu }}{{T^{2} \left( {\pi^{2} \nu^{2} + \frac{{1}}{4}\left( {2\pi \lambda - \cot \theta } \right)^{2} } \right)}}}} \left( {2\pi \lambda - \cot \theta } \right)\csc^{2} \theta }}{{\left( {\pi^{2} \nu^{2} + \frac{{1}}{4}\left( {2\pi \lambda - \cot \theta } \right)^{2} } \right)^{\frac{3}{2}} }}\left[ {\frac{{T^{2} \left( { - \pi^{2} \nu^{2} - \frac{{1}}{4}\left( {2\pi \lambda - \cot \theta } \right)^{2} + 4\pi^{3} n^{2} \nu } \right)}}{{\pi^{2} \nu^{2} + \frac{{1}}{4}\left( {2\pi \lambda - \cot \theta } \right)^{2} }}} \right]$$

Now setting

$$\frac{dY}{{d\theta }} = 0$$

We have

3.13 $$\frac{\pi }{{4T^{3} }} \frac{{e^{{\frac{{ - 2\pi^{3} n^{2} \nu }}{{T^{2} \left( {\pi^{2} \nu^{2} + \frac{{1}}{4}\left( {2\pi \lambda - \cot \theta } \right)^{2} } \right)}}}} \left( {2\pi \lambda - \cot \theta } \right)\csc^{2} \theta }}{{\left( {\pi^{2} \nu^{2} + \frac{{1}}{4}\left( {2\pi \lambda - \cot \theta } \right)^{2} } \right)^{\frac{3}{2}} }}\left[ {\frac{{T^{2} \left( { - \pi^{2} \nu^{2} - \frac{{1}}{4}\left( {2\pi \lambda - \cot \theta } \right)^{2} + 4\pi^{3} n^{2} \nu } \right)}}{{\pi^{2} \nu^{2} + \frac{{1}}{4}\left( {2\pi \lambda - \cot \theta } \right)^{2} }}} \right] = 0$$

From **(3.13)**, we see that

$$e^{{\frac{{ - 2\pi^{3} n^{2} \nu }}{{T^{2} \left( {\pi^{2} \nu^{2} + \frac{{1}}{4}\left( {2\pi \lambda - \cot \theta } \right)^{2} } \right)}}}} \ne 0\quad {\varvec{and}} \quad\csc^{2} \ne 0 on 0 < \theta \le \frac{\pi }{2}$$

Therefore

$$2\pi \lambda - \cot \theta = 0\quad {\varvec{and}}\quad T^{2} \left( { - \pi^{2} \nu^{2} - \frac{{1}}{4}\left( {2\pi \lambda - \cot \theta } \right)^{2} } \right) + 4\pi^{3} n^{2} \nu = 0$$

Now

$$2\pi \lambda - \cot \theta = 0\quad {\varvec{gives}} \quad\theta = \tan^{ - 1} \left( {\frac{{1}}{2\pi \lambda }} \right) = \theta_{opt} .$$

and

$$T^{2} \left( { - \pi^{2} \nu^{2} - \frac{{1}}{4}\left( {2\pi - \cot \theta } \right)^{2} } \right) + 4\pi^{3} n^{2} \nu = 0\quad {\varvec{gives}}\quad \left| n \right| = \frac{T}{4\pi } \sqrt {\frac{{4\pi^{2} \nu^{2} + \left( {2\pi \lambda - \cot \theta } \right)^{2} }}{\nu \pi }}$$

From **(3.13),** we see that

$$\frac{dY}{{d\theta }} > 0 when 2\pi \lambda - \cot \theta > 0\quad {\varvec{for}}\quad \left| n \right| > \frac{T}{4\pi }\sqrt {\frac{{4\pi^{2} \nu^{2} + \left( {2\pi \lambda - \cot \theta } \right)^{2} }}{\nu \pi }} .$$

This gives

$$\theta > \tan^{ - 1} \left( {\frac{1}{2\pi \lambda }} \right) = \theta_{opt}\quad \user2{ for}\quad \left| n \right| > \frac{T}{4\pi }\sqrt {\frac{{4\pi^{2} \nu^{2} + \left( {2\pi \lambda - \cot \theta } \right)^{2} }}{\nu \pi }.}$$

Again from **(3.13)** we see that

$$\frac{dY}{{d\theta }} < 0 when 2\pi \lambda - \cot \theta \left\langle\quad {0 {\varvec{for}}\quad \left| n \right|} \right\rangle \frac{T}{4\pi }\sqrt {\frac{{4\pi^{2} \nu^{2} + \left( {2\pi \lambda - \cot \theta } \right)^{2} }}{\nu \pi }} .$$

This gives

$$\theta \left\langle {\tan^{ - 1} \left( {\frac{1}{2\pi \lambda }} \right) = \theta_{opt} \quad\user2{ for }\quad \left| n \right|} \right\rangle \frac{T}{4\pi }\sqrt {\frac{{4\pi^{2} \nu^{2} + \left( {2\pi \lambda - \cot \theta } \right)^{2} }}{\nu \pi }.}$$

We proved the theorem for $\left| {C_{n,\theta } } \right|^{2}$, consequently, it holds for

$$\mathop \sum \limits_{\left| n \right| \ge N + 1 } \left| {C_{n,\theta } } \right|^{2} .$$

Therefore

$$\|u - S_{{N,\theta}}\|_{L_{2}}^{2} = \mathop \sum \limits_{\left| n \right| \ge N + 1 } \left| {C_{n,\theta } } \right|^{2}$$

Hence

$${\mathop{\hbox{arg min}}\limits_{{0 < \theta \le \frac{\pi }{2}}}}\| u - S_{{N,\theta }}\|_{L_{2}} = \theta_{opt}$$

## Conclusion

To sum up, the objectives we obtained in this work are listed below:

- Utilizing fractional Fourier series and fractional Fourier transform, we looked at the characteristics of fractional Fourier coefficients of linear chirps.
- We demonstrated that the ${\varvec{\theta}}_{{{\varvec{opt}}}}$ approach outperforms the conventional Fourier series method in the linear chirps approximation by setting the fractional Fourier parameter to a constant value.
- The analysis has discovered that using the optimal fractional Fourier parameter ${\varvec{\theta}}_{{{\varvec{opt}}}}$, zero-degree fractional Fourier coefficients attain maximum, and consequently, large-degree fractional Fourier coefficients have the fastest decay.
- We have demonstrated that the fractional Fourier series is helpful for chirp analysis and achieves minimal mean square error in the fractional Fourier parameter domain.

## Data Availability

All the data are clearly available in the manuscript.
